# The Apapacho Violence Prevention Parenting Program: Conceptual Foundations and Pathways to Scale

**DOI:** 10.3390/ijerph19148582

**Published:** 2022-07-14

**Authors:** Jorge Cuartas, Helen Baker-Henningham, Andrés Cepeda, Catalina Rey-Guerra, Instituto Colombiano de Bienestar Familiar (ICBF) Early Childhood Team

**Affiliations:** 1 Harvard Graduate School of Education, Harvard University, Cambridge, MA 02138, USA; 2 Department of Psychology, Universidad de los Andes, Bogotá 111711, Colombia; 3 School of Psychology, Bangor University, Bangor LL57 2DG, UK; h.henningham@bangor.ac.uk; 4 Caribbean Institute for Health Research, University of the West Indies, Kingston BB11000, Jamaica; 5 School of Social Work, University of Michigan, Ann Arbor, MI 48109, USA; acepeda@umich.edu; 6 Lynch School of Education and Human Development, Boston College, Chesnut Hill, MA 02467, USA; reyguerr@bc.edu

**Keywords:** violence against children, violence prevention, parenting programs, complex interventions, early childhood, Colombia

## Abstract

Violence against children (VAC) is a major global issue with long-lasting negative consequences on individuals and societies. The present study presents a review of the literature on drivers of VAC and the core components of evidence-based violence prevention programs. Moreover, it analyzes the existing services and social infrastructure in Colombia to rigorously inform the design of the Apapacho violence prevention parenting program for families with children younger than five targeted toward Colombia. Findings indicate that (1) VAC in Colombia is a multidimensional issue with roots at the individual, family, community, and society levels, (2) evidence-based violence prevention programs share a common set of content and delivery strategies that could inform the components of the Apapacho program, and (3) there is an urgent need for scalable and flexible violence prevention programs for families with young children in Colombia. Considering existing evidence, the Apapacho violence prevention parenting program will be designed using ecological, developmental, and neuroscience-informed perspectives. This article concludes by presenting the initial components of the theory of change and discussing future directions for the design of the Apapacho program and other violence prevention interventions in LMICs.

## 1. Introduction

Violence against children (VAC) is a major human rights and public health issue that can lead to long-lasting negative consequences on children’s neural and skill development, physical and mental health, and well-being [1,2,3,4]. Globally, violent punishment (euphemistically called violent “discipline”) inflicted by mothers, fathers, and other main caregivers in the home is the most common form of violence against children younger than five [5,6,7]. Indeed, more than 220 million—or about 2 out of 3—children aged 2–4 years living in low- and- middle-income countries (LMICs) experienced physical punishment or psychological aggression in the home before the COVID-19 pandemic [8,9]. Recent evidence indicates that VAC, including violent punishment, increased amid the pandemic [10]. As such, there is an urgent need for effective prevention strategies to protect young children from violence, especially in LMICs and conflict-affected settings where VAC is most prevalent [5,7,8].

The purpose of this article is to present the conceptual foundations and design plan for the Apapacho (which means “to hug the soul” in the Nahuatl language) parenting program to prevent VAC during early childhood targeted toward Colombia, following guidance on the development of complex interventions. The specific objectives of the article are to (1) identify the core components of evidence-based parenting programs that have been effective in preventing VAC in LMICs, (2) understand the context of Colombia, including the drivers of VAC and existing services, programs, and social infrastructure related to violence prevention and parenting more broadly in the country, and (3) combine the core components and context to present an initial theory of change of the Apapacho program.

### 1.1. The Colombian Context

The initial design of the Apapacho program will target Colombia. Colombia is an upper-middle income country with more than 48.2 million inhabitants, of which 8.4% are children younger than five. The country has endured a civil conflict for more than 50 years, which led to direct victimization and forced internal displacement of about 10% of its population. Regarding VAC, Save the Children recently ranked Colombia as the fifth country where children suffer the most from conflict and fourth regarding child homicide rates [11]. Furthermore, Colombia is one of the countries with the highest rates of violent punishment in Latin America, with more than 1.7 million children younger than five experiencing physical punishment before the pandemic [12]. The prevalence of physical punishment was higher in poor households as measured by the multidimensional poverty index, in rural areas, and in settings more affected by the civil conflict and contextual violence [12,13].

In May 2021, the Colombian National Congress approved a national law prohibiting all forms of violent punishment (including physical punishment and psychological aggression) in all settings, including the home. The Government also launched a national plan to reduce the high prevalence of VAC [14]. The national plan includes, among others, the objective of “creating, strengthening, or designing services, programs, and strategies aimed at preventing physical punishment and cruel, humiliating, or degrading treatment of children in all settings […]” (author’s translation) [14] (p. 51). In fact, despite some small-scale pilots in small communities [15], there are no universal programs explicitly aimed at preventing violence against young children in the home or by household members, and there is little evidence of effective strategies to prevent VAC more broadly in Colombia.

### 1.2. Violence Prevention Parenting Programs

Parenting programs were identified as key strategies to promote the nurturing care of young children and prevent VAC [7,16,17], and are expanding across LMICs in recent years [18]. Parenting programs are social and behavioral interventions aimed at improving caregiver knowledge, self-efficacy, attitudes, well-being, and daily behaviors and interactions with children [16,18]. These programs vary according to their main emphasis, including programs that primarily focus on promoting child development through early learning activities (e.g., The Reach Up program [19]) and others that emphasize content on preventing child behavior problems or violent punishment. Meta-analyses show that parenting programs focusing on psychosocial stimulation were effective in improving parenting knowledge, practices, and child development in LMICs [18], whereas parenting interventions focusing on violence prevention reduced VAC and risk factors for VAC (e.g., positive attitudes towards physical punishment) and increased positive parenting behaviors [20,21]. While psychosocial-stimulation parenting programs were evaluated extensively in LMICs [18], most evidence on violence prevention parenting programs comes from high-income countries (HICs), and less is known about the design, implementation, and effectiveness of these interventions in LMICs [20,21,22]. Research on violence prevention parenting programs grew in the past five years [23,24,25,26], but there is still a need for further research on the design, implementation, and effectiveness of these type of interventions in LMICs.

### 1.3. Core Components and the Development of Complex Interventions

Researchers, practitioners, and policymakers sometimes transported and, in some cases, adapted violence prevention parenting programs developed in HICs to LMICs, such as Incredible Years [27] and Triple P [28,29]. However, there is scarce and rather mixed evidence on the transportability of parenting programs across countries and cultures [30,31,32]. Furthermore, the vast variability in languages, cultures, social norms, and social and policy infrastructures between and within countries might make interventions transported from HICs to LMICs irrelevant or inadequate [33,34] Moreover, there are limits to the extent to which interventions can be adapted to meet the needs/culture of families without becoming an entirely different intervention. There are other disadvantages and barriers to transporting existing interventions, including access restrictions, barriers to discarding irrelevant content, difficulty of implementing with fidelity and embedding in existing social infrastructure, and implementation costs (e.g., licensing, material production, training, ongoing consultation with developers) that might be prohibitively high for LMICs [35].

An alternative to fully transported or homegrown interventions is adapting “core components” of evidence-based parenting programs to the context of interest. Core components—or “active ingredients”—are fundamental principles and procedures that are thought to underlie the effectiveness of a given intervention [35,36]. Recently, some violence prevention programs were developed using similar approaches, including Parenting for Lifelong Health [37] and The Irie Homes Toolbox [38].

Using evidence-based core components as a starting point, instead of starting from scratch or fully transporting an intervention, has at least three main relative advantages. First, identifying and using core components allows intervention developers to use all existing evidence to “distill” best-practice recommendations, instead of just employing the components from a single intervention or starting from scratch. Second, adapting core components, instead of transporting an intervention, can improve implementation outcomes, including acceptability, adoption, appropriateness, feasibility, fidelity, integration, and sustainability [39] (see Table 1), as well as the effectiveness of the intervention. Finally, this approach facilitates the use of dynamic adaption that results in a more flexible and scalable intervention, following best-practice recommendations to develop complex interventions [40,41].

Guidance on how to develop complex interventions comprises four major phases [40,41]. First, a preparation phase, which involves defining the problem, planning the development process, and engaging key stakeholders (e.g., who will deliver and benefit from the intervention) [42]. Second, a conceptualization phase, including reviewing published evidence on the core components of evidence-based interventions, drawing on existing theories, understanding the context, and drafting the initial theory of change. Third, an integration and rapid learning phase that includes formative research, preliminary content design, and iterative piloting in the context. Finally, a completion phase, including conducting feasibility studies. In this manuscript, we report findings from the first and second phases of the design of the Apapacho program.

### 1.4. The Present Study

We respond to policy calls on the need for VAC prevention parenting programs in Colombia [14] by working on the design of the Apapacho violence prevention parenting program, a scalable program for families with children younger than five. We will design the Apapacho program in a participatory fashion, working directly with Colombian policymakers and staff from the Instituto Colombiano de Bienestar Familiar [Colombian Institute of Family Welfare] (ICBF, for its acronym in Spanish), which is the public institution in charge of the promotion and protection of early childhood, childhood, adolescent, and family policy and services in Colombia. In this article, we review the literature on the drivers of VAC and the core components (e.g., theoretical basis, contents, implementation strategies) of evidence-based violence prevention parenting programs for families with young children. Furthermore, we present a description of the existing social services and programs related to violence prevention and parenting in Colombia to identify pathways to scale. Doing so, this study describes the development of the conceptual foundation that will inform the design of an evidence-based, scalable violence prevention program for Colombia.

## 2. Materials and Methods

We used three sources of information for this study. First, we searched PsycInfo (EBSCO), PubMed, and Google Scholar for literature on the theoretical basis and core components of programs to prevent violence against children and/or child behavior problems. We identified systematic reviews and meta-analyses of global evidence, as well as programs implemented in LMICs with experimental evidence. We focused on programs from LMICs to assess whether their core components are consistent with the core components identified in reviews. We searched for publications, facilitator manuals (when available/open), and other relevant information from the interventions implemented in LMICs. We also searched for literature on the predictors of VAC in the same sources.

Second, we searched for and examined the operational manuals of the programs and services for children younger than five and their families provided by the ICBF. Finally, we complemented this review of operational manuals with conversations with staff from the ICBF to map all existing programs and services related to early childhood, families, and violence prevention in Colombia (the team who participated in these conversations are coauthors in this manuscript).

This study received ethical approval from the Institutional Review Board (IRB) at Harvard University on 11 April 2022 (IRB Registration # IRB00000109) and received technical approval from the ICBF on 25 January 2022.

## 3. Results

### 3.1. Core Components of Evidence-Based Parenting Programs

We found six recent reviews and meta-analyses of parenting programs for caregivers of young children to prevent violence and child behavior problems [43,44,45,46,47,48]. Furthermore, we found 17 recent randomized controlled trials of 11 parenting programs implemented in LMICs targeted toward young children, which were effective in reducing VAC, key drivers of VAC, and child behavior problems (see Table 2) [15,23,24,25,26,28,29,49,50,51,52,53,54,55,56,57,58].

Overall, global reviews and the 11 programs suggest a common set of core components that might be relevant for violence prevention parenting programs. First, most programs use a cognitive–behavioral and/or social learning theoretical perspective. Second, most programs include content to promote (i) knowledge of children’s development and needs, (ii) caregiver engagement in play and other learning activities, (iii) emotional communication skills, and (iv) use of discipline strategies (e.g., positive reinforcement, natural/logic consequences, and consistent limit setting). Third, programs often include a focus on caregivers’ self-care, well-being, and stress management, for example, by introducing self-regulation techniques and less commonly dealing with a history of adversity (e.g., violence in childhood or intimate partner violence). Finally, some programs include content to inform caregivers about the effects of violent punishment, e.g., [26], and one program includes explicit content to promote fathers’ engagement in caregiving [49,50].

Regarding delivery techniques, programs were delivered using group meetings, home visits, and a combination of both. Key components also include delivering content in positive, non-stigmatizing ways using positive and supportive feedback from facilitators, group discussion, and peer support to bolster caregiver self-confidence. Moreover, most programs used participatory and experiential approaches to promote knowledge and skills, including demonstrations and modeling from trained facilitators, role-playing and rehearsal/practice of behaviors, and homework to practice skills at home. Finally, most programs promote setting parenting goals and reviewing such goals and provide materials (e.g., toys, books, and other printed materials).

#### Main Takeaway

Programs that were effective in preventing and reducing VAC and addressing drivers of VAC have shared core components, including content and delivery strategies, that could inform the development of the Apapacho violence prevention parenting program. Relevant content comprises knowledge and attitudes (for example, related to child development and parenting), emotional self-regulation (including emotion knowledge and regulation), family relationships (including emotional communication), stimulation in the home (including child-led play), and adequate discipline techniques (including positive reinforcement and prevention of child behavior problems), among others. Regarding delivery, most programs used highly interactive and participatory strategies.

### 3.2. Drivers of VAC in Colombia

Global research, including reviews [7,59,60] and cross-cultural studies [61,62], showed that multiple factors at the individual, family, community, and society levels predict VAC. Some of these factors include caregivers’ beliefs that children need physical punishment to be raised properly, caregivers’ mental health and self-regulation skills, caregivers’ own exposure to physical punishment in their childhood, low caregiver education, lower socioeconomic status, living in a violent context, living in rural vs. urban areas, and gender inequity, among others. Several studies also conclude that children aged 3–5 years old are at higher risk of experiencing physical punishment relative to younger children, and there are mixed findings related to sex-related differences [59,61].

**Figure 1:**
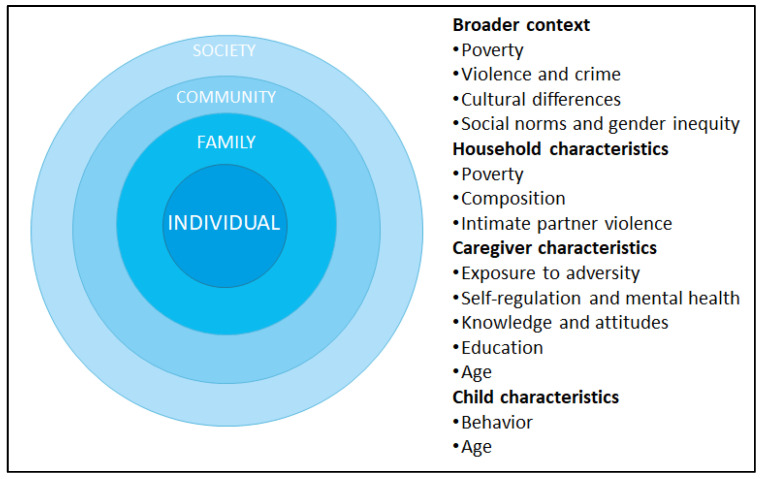
Key drivers of VAC in Colombia.

We identified three studies of the drivers of VAC during early childhood—physical punishment specifically—in Colombia, using data from nationally representative surveys [12,13,63]. In general, these studies obtained results that are mostly consistent with evidence from global research (Figure 1). First, at the child level, these studies did not find differences by sex, but children aged 2–5 years old were more likely to experience physical punishment than younger children. Yet, in every region at least 10% of children were physically punished, even before their first birthday [12]. At the family level, physical punishment was more common when the caregiver was older, if the father or partner of the mother lived in the household, and if the mother had the child when she was a teenager. Other family-level risk factors included caregivers’ own exposure to physical punishment in childhood, positive attitudes to domestic violence (e.g., justifications for violence against children), having a higher ratio of children per total household members, and household poverty.

At the broader context, physical punishment was more common in neighborhoods with higher violent crime rates (e.g., homicides), in regions with higher levels of poverty and more exposed to the civil conflict (as measured by homicides rates and presence of guerrillas), and in rural areas [13,63]. Finally, the studies identified substantial regional variability across the country, and differences according to ethnicity and within different minority groups (e.g., afro-Colombians and indigenous communities), possibly indicating cultural differences and variation in social norms across regions and communities [13].

#### Main Takeaway

Collectively, global research and evidence from Colombia support a social–ecological perspective [7,64,65], indicating that VAC is a multidimensional problem with drivers at the individual, family, community, and society levels. Yet, most research suggests that these risk factors might impact the risk of VAC through their effect on caregiver cognitions (e.g., knowledge and skills related to child behavior and appropriate discipline; beliefs and attitudes toward VAC) and emotions (e.g., stress and mental health). These are key outcomes that parenting programs often seek to address, according to our review of core components.

### 3.3. Pathways to Scale: Current Services and Infrastructure in Colombia

The ICBF is the entity of the Colombian state with responsibility for services for children and families, including child education and child protection from early childhood through to adolescence. The ICBF has a nationwide coverage but prioritizes at-risk families (e.g., experiencing poverty or with past cases of child maltreatment), and currently serves more than 8 million Colombians. The ICBF has six different divisions, but in the remainder of this section we focus on the Dirección de Primera Infancia (i.e., early childhood division) and Dirección de Familias y Comunidades (i.e., families and communities division), as they are responsible for services targeting early childhood, families, and the promotion of positive parenting.

#### 3.3.1. Services for Young Children

According to internal administrative data from the ICBF, the early childhood division serves around 1.7 million children younger than five and their families through four modalities of service provision, namely Institutional (approximately 461,525 children), Family (661,100), Community (473,599), and Ethnic and Intercultural modality (95,306). Table 3 presents an overview of the target population and the main services of each attention modality.

The objective of the Institutional modality is to promote early childhood development through holistic and structured early childhood care and education services [66]. Its target population includes children between ages two and five years, but it also serves children younger than two in special circumstances. The Institutional Modality has six main services (see Table 3). Its flagship service is Child Development Centers (CDI, for its acronym in Spanish). CDIs provide care and education in specialized ECD centers five days per week, 8 h per day, nationwide but mainly in urban areas. CDIs are typically staffed with an interdisciplinary team comprising teachers, psychologists, social workers, and nutritionists. CDIs have expanded substantially throughout Colombia since 2010, exhibiting positive impacts on child developmental outcomes [67], but still have heterogeneous (ranging from low to moderate) structural and process quality [68].

The Family modality seeks to promote early childhood development and positive parenting at home, prioritizing children and families in rural areas and who do not have access to other early education and care services [69]. The target population are children from birth to five years of age. The Family modality’s main services are family-based care (DIMF, for its acronym in Spanish), Rural Early Childhood Education Service (EIR), and Family, Women, and Infancy Community Welfare Homes (HCB FAMI). These services are delivered through group sessions, home visits, and through radio and delivery of materials (in EIR). The services are based on a framework of 17 care and child-rearing practices that are adapted according to the particularities and needs of each family and community. The group sessions take place in community spaces, such as schools, churches, or the ECD teachers’ homes. Each teacher works with a group of 12–15 families. The EIR service was designed on a multimodal scheme that includes telephone-based support and educational radio. All the services of this modality include an interdisciplinary ECD team. Family modality’s services do not have specific curricula, apart from some basic operational guidelines and a general list of contents that facilitators are responsible for developing and implementing [70]. A recent experimental evaluation found that introducing a structured curriculum to the HCB FAMI was effective in improving child outcomes and the quality of the home environment [70].

The Community modality is a care and education service, which children attend every day of the week for about 6–8 h [71]. This modality targets children between ages 18 months and 5 years from both urban and rural areas. Its main service is Community welfare homes (HCB), which is early childhood care arrangement that provides childcare, nutrition, and stimulation in the homes of community mothers or fathers for 10–14 children each. There are no explicit curricula, besides basic operational guidelines, for the provision of this service. One non-experimental evaluation found associations between the length of exposure to HCB and children’s gains in cognitive and social-emotional development [72].

Finally, the Ethnic and Intercultural modality was developed taking into account local traditional and indigenous perspectives to include social participation and cultural factors within service provision [73,74]. This modality targets children younger than five years from ethnic and rural communities and seeks to provide early childhood care and education and support families, while respecting cultural traditions, territorial particularities, and the local, social, and political structure. An intercultural team leads service provision using community spaces and families’ homes.

The early childhood division leads other initiatives targeting the prevention of VAC, including programs (1) to identify risk factors and actual cases of VAC in early care and education services (program Guardianes de la Niñez [Guardians of Childhood]), (2) to train ICBF staff, teacher and multidisciplinary teams in the detection and response to VAC, (3) to strengthen ICBF staff, teacher and multidisciplinary teams’ socioemotional skills [Sanar para Crecer], and (4) to provide ongoing support to families amid the COVID-19 pandemic (Mis Manos te Enseñan [My Hands Teach You] strategy).

#### 3.3.2. Other Services for Families and Communities

Besides services that target young children, the ICBF provides services for families with children and adolescents. One such service is Mi Familia (i.e., My Family), an indicated parenting psychosocial program that seeks to strengthen parenting capabilities to promote child and adolescent development and guarantee their protection [75]. The program targets families with prior cases of abuse and/or neglect. Family Accompaniment Professionals (PAF, for its acronym in Spanish) implement Mi Familia through home visits and family meetings. There are more than 2900 PAF, each supporting 10–22 families.

For families with children younger than five, Mi Familia comprises nine home visits and four family meetings. In each meeting, the PAF follow methodological booklets to implement the session content. Broadly, the booklets include content on self-care, emotional regulation, assertive communication, conflict solving in the home, and positive parenting. A key feature of Mi Familia is that it is highly flexible and each PAF works with families to design an intervention plan that responds to families’ needs. To date, Mi Familia has not been evaluated.

#### 3.3.3. Main Takeaway

The ICBF has several different modalities and uses multiple delivery platforms to respond to different needs, territorial particularities, and cultures. To date, there are several universal programs aimed at promoting young children’s development and caregiver positive-parenting practices. Yet, there are fewer programs aimed at preventing VAC in the home, and besides notable indicated (i.e., targeting at-risk families) programs, such as Mi Familia, there are no universal or selective programs with experimental evidence and clear theories of change and/or curricula aimed at preventing VAC. Considering that there is a solid social/service infrastructure and prior successful experience of integrating new curricula to improve service provision [70], there are opportunities to design, implement, and scale curricula targeting the prevention of violence against young children.

### 3.4. Putting It All Together: Conceptual Basis and Objectives of the Apapacho Violence Prevention Parenting Program

In this section, we combine evidence on the core components of parenting programs that were effective in preventing or reducing VAC, information on the drivers of VAC in Colombia, and the current services and infrastructure to support early childhood development and prevent VAC in Colombia to propose the conceptual foundations and objectives of the Apapacho program.

#### 3.4.1. Ecological Perspective

We will employ a bioecological perspective [64,65] as an overarching framework in the development of the Apapacho program. A bioecological perspective posits that reciprocal interactions between individual, family, community, and social factors influence both children’s development and their protection vs. vulnerability or risk of experiencing violence. This perspective also recognizes that experiences and context influence the knowledge, attitudes, beliefs, self-confidence, and behaviors of mothers, fathers, and other adult caregivers. Reciprocity is a key feature of this perspective: for example, caregiver physical punishment contributes to children externalizing behaviors, and children externalizing behaviors can also elicit impulsive/violent reactions from caregivers if they do not have the knowledge and skills to provide appropriate responses, hence producing a vicious cycle of aggression.

A bioecological perspective allows us to operationalize some of the drivers of VAC in the Colombian context. First, some of the factors identified in previous research are proximal, malleable risk factors that could be targeted through a parenting program, such as Apapacho, including caregiver knowledge, attitudes, beliefs, self-regulation, and self-confidence. Second, other risk factors comprise past or ongoing experiences that require attention in the intervention content, including a history of violence in caregiver childhood or intimate partner violence. Third, some factors are more structural and could inform the targeting of the intervention, including household poverty, low caregiver education, teenage pregnancy, and high levels of contextual violence (crime or presence of armed groups). Finally, some risk factors suggest that the intervention must be designed to be sensitive to specific dynamics and needs, including family composition, the presence of fathers in the home, and regional and cultural differences within Colombia, among others.

#### 3.4.2. Developmental Perspective

A developmental perspective indicates that early childhood is a sensitive period of development, when rapid brain and skill development occurs in response to experience [76]. During these years, relationships are the main catalyst for development and adult caregiver behaviors have a significant impact on child skill development and behaviors (e.g., harsh parenting could contribute to child behavior problems) [17,77,78]. In the first years of life, caregiver engagement in child-led play and other stimulation activities also exert an important influence on the development of critical skills, including executive function and self-regulation skills [79,80].

A developmental perspective also highlights the need for the Apapacho program to be sensitive to the different needs and challenges that both parents and children experience throughout the first years of life (see Table 4). For instance, understanding children’s cues is a challenge caregivers face in infancy, before children develop language. This perspective is consistent with evidence on the drivers of VAC in Colombia, which indicates that the risk of experiencing VAC varies with children’s age [12,61].

#### 3.4.3. A Neuroscience-Informed Perspective on Adult and Child Core Capabilities

Traditional models of training and support for caregivers largely rely on providing information (e.g., on the negative consequences of physical punishment), which could not meet the needs of caregivers, in particular of those facing past or ongoing adversity (i.e., poverty or violence), and may not be effective in helping caregivers develop the skills they need for parenting [86]. Extensive evidence about the drivers of VAC and the core components of parenting programs indicates that supporting caregiver knowledge, attitudes, and self-regulation skills is critical to prevent VAC. This is consistent with contemporary neuroscience-informed perspectives on how to support adults and children build core capabilities [86,87]. These core capabilities include self-regulation and executive function skills, which allow both adults and children to have self-control, plan, direct and sustain attention/focus, and be flexible [86,87,88]. These core capabilities are increasingly recognized as fundamental to build other skills (e.g., cognitive skills), and to cope with challenges, including the daily challenges of parenting [87].

The development of the Apapacho program will use this neuroscience-informed approach to promote caregivers’ core capabilities to prevent and reduce VAC. We hypothesize that such an approach can increase the effectiveness of Apapacho for three reasons. First, there is evidence that the core skills that parents and caregivers require for parenting (e.g., abilities to self-regulate emotions and behaviors, plan, attend to children’s cues, and be flexible) can be strengthened with practice [86,88,89]. Second, child self-regulation and executive function skills can also reduce child behaviors that can be interpreted as challenging or difficult by caregivers (e.g., tantrums) [90]. Finally, the development of these skills can help adults and children who have experienced past or ongoing violence by supporting the development of resilience and coping skills [17,91,92]. As such, this perspective takes a strength-based approach to building core capabilities for adults and children to promote positive parenting and child protection.

#### 3.4.4. Objectives of the Apapacho Violence Prevention Parenting Program

Considering these conceptual foundations, the main objectives of the Apapacho violence prevention parenting program are to:

1. Prevent violence against children younger than five by parents/caregivers;
2. Promote caregivers’ core capabilities for parenting (including knowledge, sense of competence, positive attitudes, adequate expectations, executive functions, and self-regulation) and daily positive-parenting practices (including play, other forms of stimulation, and developmentally appropriate discipline);
3. Promote young children’s executive function and self-regulation skills to support their overall development.

#### 3.4.5. Initial Components of the Theory of Change

**Figure 2:**
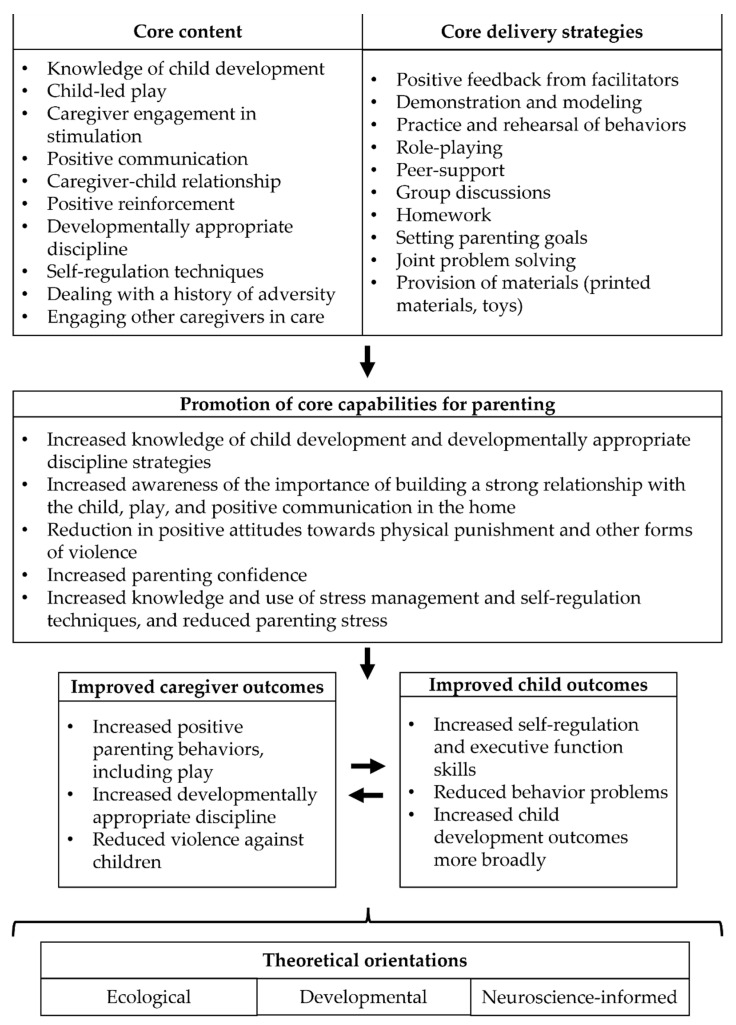
Initial components of the theory of change of the Apapacho violence prevention parenting program.

Apapacho will be a parenting program based on ecological, developmental, and neuroscience-informed perspectives to prevent and/or reduce violence against children younger than five by promoting caregivers’ core capabilities for parenting and children’s executive function and self-regulation skills (see Figure 2). To achieve these objectives, Apapacho will encompass content and delivery strategies identified as core components of parenting programs that have been effective in preventing VAC, including building knowledge of child development and discipline techniques, promoting play and learning activities, communication skills, caregivers’ emotion knowledge and regulation, and delivering content using interactive and participatory strategies, among others. These components will be adapted in processes of rapid-piloting co-design with key stakeholders (policymakers, caregivers, potential program facilitators) to improve implementation outcomes considering the existing infrastructure of the ICBF and other contextual factors in Colombia.

## 4. Discussion

This manuscript describes the conceptual foundations of the Apapacho violence prevention parenting program targeted toward Colombia. We developed these foundations following recent guidance on how to develop complex interventions [40,41], integrating evidence on the core components of violence prevention parenting programs with information about the drivers of VAC and existing services and social infrastructure in Colombia. We developed an initial theory of change based on theory, evidence, and context, proposing the core content and delivery strategies that the Apapacho program will use to promote core capabilities for parenting, improve caregiver outcomes (e.g., increased positive behaviors and reduced violence against children), and improve child outcomes (e.g., increased self-regulation and executive function skills). Following the guidance on developing complex interventions, the next step in this work will be to adapt these components to the local context using qualitative evidence from key stakeholders’ (e.g., policymakers, parents, facilitators) attitudes, beliefs, and perceptions, as well as iterative processes of rapid-cycle piloting to improve implementation outcomes and potential effectiveness.

The Apapacho program will incorporate content to promote parenting knowledge, positive attitudes, self-regulation, and well-being, among others. In general, this content seeks to build core capabilities for parenting [86] and, in particular, influence cognitive (e.g., knowledge, attitudes, confidence) and emotional factors (i.e., self-regulation, satisfaction) that are thought to be proximal drivers of parenting practices and behaviors [82,89,93,94]. For instance, parenting knowledge provides information for caregivers on how to respond to specific children’s needs or behaviors during different periods of development. Similarly, attitudes and norms provide a framework for caregivers to determine behaviors that are justified or needed to raise a child, making it more likely that caregivers use such behaviors. As a final example, self-regulation skills allow caregivers to regulate their emotions and behaviors in the face of challenges (e.g., tantrums), inhibiting automatic responses (e.g., spanking the child or yelling), and responding instead in ways that are developmentally appropriate.

The Apapacho program will also include delivery approaches based on experiential, participatory, and highly interactive strategies, including group discussions and support, demonstrations, practicing, joint problem-solving, and homework. These approaches are considered critical components of effective parenting programs in LMICs, e.g., [37,38] and are consistent with principles of behavior change. For example, the COM-B system for understanding behavior posits that capability, motivation, and opportunity are needed for a behavior to occur. The strategies that Apapacho will use will directly influence these factors by promoting key capabilities for parenting, delivering content in ways that make the sessions enjoyable and might build parenting confidence (therefore increasing motivation), and providing opportunities for caregivers to practice and use behaviors and strategies (e.g., homework).

We used a “core components approach” to develop the conceptual foundations of Apapacho, instead of transporting an existing intervention with evidence on effectiveness. Using such an approach is advantageous in the context of Colombia, where the national plan to prevent VAC makes a call for scalable interventions that can be embedded into existing services or social infrastructure [14]. By developing the Apapacho program in a participatory fashion, adapting core components specifically for the context of Colombia, we can also maximize implementation outcomes, including its acceptability, feasibility, and integration, among others. Finally, by using this approach we can develop an open-access intervention, reducing issues of high licensing costs or requirements of highly qualified facilitators, which might not be available in several regions of Colombia.

The core components approach also allows us to develop a flexible intervention that responds to the specific needs and social infrastructure of Colombia and can be integrated into the different service modalities operated by the ICBF, maximizing its feasibility and scalability. Indeed, prior studies demonstrate that such an approach could strengthen existing services from the ICBF and have positive impacts on families and children. For example, a study showed that embedding a structured early stimulation curriculum into the existing HCB FAMI improved child development outcomes and maternal knowledge about child development, among other outcomes [70]. Therefore, embedding an intervention aimed at preventing VAC can strengthen existing services and reach all families being served by the ICBF.

This article has some limitations that inform future directions for research. First, the article relies mostly on secondary data, so future studies should collect primary information to better understand families’, policymakers’, and potential facilitators’ needs and perceptions to adapt the core components. We will do this by collecting qualitative data and conducting rapid-cycle pilots in a process of ongoing adaptation and improvement. Such an iterative process will allow us to have an intervention that is more relevant to the local culture, context, and social infrastructure and that could be evaluated to inform its potential scalability. Second, there was scarce evidence on the drivers of VAC in Colombia, so additional work is needed to identify other factors that might inform the content or targeting of Apapacho and other violence prevention programs. Finally, this article focused on Colombia, but there is growing need for violence prevention parenting programs in other LMICs. Nevertheless, the rigorous identification of core components for the Apapacho program will inform future studies aimed at designing or adapting parenting programs in other LMICs.

## 5. Conclusions

VAC is prevalent in LMICs and can undermine young children’s health and development. To date, most interventions to prevent VAC in the home were developed and evaluated in HICs, but there is a growing number of studies from LMICs in the last few years, e.g., [37,38,49]. In this study, we directly respond to a gap in knowledge and policy calls [14] and we present the conceptual foundations of the Apapacho violence prevention parenting program targeted toward Colombia. The initial components of the Apapacho program inform future steps for the design of the program, as well as future research aimed at designing effective strategies to prevent VAC and promote the healthy development of young children worldwide.

## Figures and Tables

**Table 1:** Definition of implementation outcomes *.

Outcome	Definition	Expected Outputs of Using Evidence-Based Core Components as Starting Point
Acceptability	Extent to which the intervention or program is agreeable or satisfactory to key implementation stakeholders	The development of the intervention is more flexible and can respond to local needs, preferences, and culture, ensuring acceptability
Adoption	Initial intention, decision, or action to implement the intervention or program	The interventions can be developed with active participation of key stakeholders to ensure future adoption
Appropriateness	Refers to the fit, relevance, or compatibility of the intervention or program for a specific setting, culture, social service or infrastructure, implementation stakeholder, user, and for addressing the unmet need or problem	Core components can be adapted through qualitative and quantitative research and rapid-learning cycles to the specific setting, culture, and social infrastructure, maximizing its appropriateness
Feasibility	Extent to which the intervention or program can be successfully employed or implemented by the implementation stakeholders within a particular social infrastructure	Combining core components with an in-depth knowledge of the local institutional infrastructure allows adapting these components to fit existing services, social infrastructure, and implementation capacity, increasing its feasibility
Fidelity	Degree to which the intervention or program is implemented as planned in the original protocol or design	Core components can be adapted to meet the local implementation capacity (e.g., characteristics of potential facilitators), making it more likely that the intervention can be implemented with fidelity
Integration	Degree to which the intervention or program integrates within an existing social service or infrastructure	Core components underlying the intervention can be adapted to fit existing social services and infrastructure, increasing the integration of the intervention
Sustainability	Extent to which an intervention or program can be maintained or institutionalized within an existing social service or infrastructure	As the intervention is designed specifically for the context, using existing services and existing staff, it is more likely to be sustainable at scale

* These definitions were adapted from Proctor and coauthors [39].

**Table 2:** Selected components of programs from LMICs with experimental evidence *.

Program	Evaluation Country	Format	Selected Key Content	Selected Key Delivery Strategies
ACT Raising Safe Kids	Brazil [26]	An initial meeting and eight 2-h group sessions, once a week for eight weeks	***Knowledge and attitudes*** Child development and children’s needs Effects of violent punishment ***Emotional self-regulation*** Understanding emotions Managing caregiver anger ***Family relationships*** Communication skills ***Discipline*** How to manage child aggression and behavior problems, including consequences, distracting the child, and consistent limit setting ***Other content*** Impact of electronic media on children	Explanations using media (slideshows and video) Role-play Case studies Play activities Group discussions
Cuna Más	Peru [58]	Weekly 1-h home visits	***Family relationships*** Positive caregiver–child interactions ***Play and other stimulation*** Child-led play Caregiver engagement in other stimulation/learning activities Improving the learning environment	Provision of materials (toys and books) Modeling/demonstration Rehearsal or behaviors Homework
Día a Día	Chile [56]	Six 2-h weekly sessions in preschool education centers	***Play and other stimulation*** Child-led play Caregiver engagement in other stimulation/learning activities Improving the learning environment ***Discipline*** Routines and transitions Prevention and response to child behavior problems, including withdrawal of attention and time out Promotion of positive behavior (e.g., praise)	Participatory analysis of content Videos showing interactions between parents and children Role-play Rehearsal of behaviors Homework
Adaptation of the International Child Development Programme	Bosnia [51] Colombia [15]	Group meetings, but different number of sessions in different countries	***Knowledge and attitudes*** Child development and children’s needs Caregiver self-confidence ***Family relationships*** Caregiver sensitiveness Positive caregiver–child interactions ***Play and other stimulation*** Spending quality time with children	Group discussion Praise and confirmation Modeling/demonstration Role-play Rehearsal of behaviors Homework
Irie Homes Toolbox	Jamaica [23]	Group meetings, 90-min sessions, once per week for eight weeks	***Knowledge and attitudes*** Child development and children’s needs ***Emotional self-regulation*** Understanding own and children’s emotions ***Family relationships*** Communication skills ***Play and other stimulation*** Child-led play Caregiver engagement in other stimulation/learning activities Promotion of children’s academic skills ***Discipline*** Prevention and response to child behavior problems, including withdrawal of attention, redirecting behavior, clear instructions, natural consequences, and time-out Promotion of positive behavior (e.g., praise, modeling desired behavior)	Modeling/demonstration Rehearsal of behaviors Positive and supportive feedback Collaborative facilitation Role-play Problem-solving Games and songs in each session Provision of materials Group support Goal setting Homework
Parenting for Lifelong Health	Philippines [53] South Africa [25,52] Thailand [24]	Group meetings, 2–3 h sessions, once per week for 12 weeks	***Knowledge and attitudes*** Child development and children’s needs ***Emotional self-regulation*** Understanding emotions Problem solving skills ***Family relationships*** Communication skills ***Play and other stimulation*** Child-led play Caregiver engagement in other stimulation/learning activities ***Discipline*** Prevention and response to child behavior problems, including instruction-giving, ignoring negative attention seeking, using consequences, time-out Promotion of positive behavior (e.g., praising, rewarding) Household rules and routines	Group discussion Role-play Modeling/demonstration Collaborative facilitation Rehearsal of behavior Problem-solving Homework Illustrated stories Ongoing supervision Parent support groups
Parents Make the Difference	Liberia [55]	Group-based sessions and one home visit, 2-h each for 10 weeks	***Knowledge and attitudes*** Child development and children’s needs Parenting goals ***Emotional self-regulation*** Parent self-care and stress management Reflecting on parents’ own childhood ***Family relationships*** Communication skills ***Play and other stimulation*** Caregiver engagement in play and learning activities ***Discipline*** Prevention and response to child behavior problems, including ignoring negative attention seeking and time-out Promotion of positive behavior (e.g., praising, rewarding) Household rules and routines	Group discussion In-session practice of skills Modeling/demonstration Problem-solving
Triple P	Indonesia [29] Panama [28]	Three 90-min seminars, once per week	***Emotional self-regulation*** Parent self-care and stress management Understanding emotions and emotion expression Resilience and coping skills ***Play and other stimulation*** Caregiver engagement in play and learning activities Promotion of children’s social skills ***Discipline*** Promotion of positive behavior (praise) Use of logical consequences	Explanations using media (slideshows) Materials, e.g., tip sheets
Projeto Parceria	Brazil [57]	16 weekly sessions, 50–60 min each	***Knowledge and attitudes*** Effects of violent punishment ***Emotional self-regulation*** Understanding emotions Dealing with a history of intimate partner violence and other traumatic experiences ***Family relationships*** Importance of valuing child’s efforts to improve ***Play and other stimulation*** Promotion of children’s social skills Discipline Prevention and response to child behavior problems, including ignoring negative attention seeking and time-out Household rules and routines Identifying appropriate behaviors	Discussion based on written materials Relaxation training Reading manual together with facilitator Role-play Homework
SOS! Help for Parents	Iran [54]	Two 2-h sessions, one per week	***Knowledge and attitudes*** Parenting skills Common mistakes in parenting ***Discipline*** Techniques to increase positive child behavior and reduce and prevent behavior problems	Discussions with trained physician Role-play Video-clips
Sugira Muryango	Rwanda [49,50]	12 home visits, 90-min each	***Knowledge and attitudes*** Child development and children’s needs ***Emotional self-regulation*** Family history and narrative Parent self-care and stress management ***Family relationships*** Communication skills Resolving conflicts in the home The importance of fathers’ engagement ***Play and other stimulation*** Child-led play Caregiver engagement in other stimulation/learning activities ***Discipline*** Prevention and response to child behavior problems, including ignoring negative attention seeking and time-out ***Other content*** Nutrition, hygiene, and health	Psychoeducation Active coaching Live feedback of caregiver–child interactions Problem-solving

* The table presents selected, not all, content and delivery strategies. We could not access materials/facilitator manuals for some programs; therefore, this summary is not intended to capture all contents and delivery strategies of each program.

**Table 3:** Attention modalities and services for early childhood *.

Modality (Children and Pregnant Women in 2022)	Approach	Target Population	Examples of Services
Institutional (461,525)	Center-based	Targets children between ages 2 to 5 years	Child Development Centers Multi-purpose community lefts Other type of lefts (e.g., infant care, company-based)
Family (661,100)	Home-based	Children younger than six years, prioritizing children younger than five, who do not have access to other modalities of early care and education, with a focus on rural areas	Family-based infant development (DIMF) Family, Women, and Infancy Community welfare homes (HCB FAMI) Rural early childhood education (EIR)
Community (473,599)	Community-based	Children between ages 18 months and 5 years from urban and rural areas	Community welfare homes (HCB) Grouped community welfare homes
Ethnic and Intercultural (95,306)	Multiple approaches	Children younger than five years from ethnic and rural communities	Household meetings Community meetings

* Information taken from internal administrative data from the ICBF.

**Table 4:** Some examples of relevant content from parenting programs that is sensitive to children’s and parents’ needs and challenges during different periods of development *.

	Some Needs and Risk Factors Related to VAC		Potential Content of Parenting Programs to Prevent VAC
	Children	Caregivers	
Childbirth	Differential prenatal and obstetric care due to child sex	Tiredness Postnatal depression Increased risk for IPV Sleep routines	Preparing parents through anticipatory guidance that promotes foundational skills related to the importance of building a strong relationship, attending to children’s cues, awareness of gender norms, self-care, and self-regulation
Infancy (<12 months)	Poor relationship with at least one caregiver Neglect Physical punishment	Same as above, plus: Responding to infant crying and fussing Understanding children’s cues and communication Early stimulation and attention	Same as above, plus: Promotion of knowledge about children’s needs and behaviors during different periods of development, management of behaviors that might be interpreted as challenging by caregivers (e.g., fussing/crying, sleep and feeding routines), and play and other early stimulation activities How to support children’s cognitive and social-emotional development
Toddlerhood (12–36 months)	Same as above, plus: More frequent physical punishment Psychological maltreatment Witnessing IPV Harmful gender norms	Same as above, plus: Management of tantrums Dealing with increased curiosity and demands from children (e.g., questions, or saying “no” on a regular basis) Children imitate behaviors Understanding children’s emergent preferences and personalities Children seek praise and approval	Same as above, plus: Establishing more structured routines and rules in the home Strengthening communication and explanations Strengthening knowledge related to normal child development and behavior and managing behaviors that might be interpreted as challenging by caregivers (e.g., tantrums)
Early childhood (3–6 years)	Same as above, plus: Increased exposure to risks in other settings (community, preschool) Higher likelihood of witnessing contextual violence Unequal learning opportunities due to sex/gender	Same as above, plus: Increased challenge of dealing with children’s questions and demands Helping children transition to school and interact more with other children and adults in different settings Increased challenge of dealing with verbal expression of frustration and other strong emotions	Same as above, plus: Supporting children’s transition to educational settings Strengthening knowledge about developmentally appropriate discipline

* Adapted from multiple sources [81,82,83,84,85].

## Data Availability

Not applicable.
